# Experimental investigation of heat transfer for diesel spray impingement on a high temperature wall

**DOI:** 10.1038/s41598-022-10959-6

**Published:** 2022-04-26

**Authors:** Zhenyao Guo, Weizheng Zhang, Shuang Jin, Zhicheng Shi, Yanpeng Yuan

**Affiliations:** School of Mechanical Engineering, Beijing Institute of Technology, Beijing, 100081 China

**Keywords:** Energy science and technology, Engineering

## Abstract

In this paper, the heat transfer characteristics of spray-wall impingement on a high temperature wall were studied by using a transient thermocouple and a one-dimensional finite-difference conduction model to obtain variations of wall temperature and heat flux. Results showed that increasing the injection pressure and decreasing the ambient temperature both caused an increase in surface heat flux and heat transfer coefficient. However, with the increase of the initial surface temperature from 200 to 600 °C, the surface heat flux and heat transfer coefficient first increased and then decreased, and reached the maximum at about 520 °C and 390 °C respectively, which was due to the change of heat transfer regime on the wall. The contribution of experimental factors descended in the order of initial surface temperature, injection pressure and ambient temperature. The dimensionless surface heat fluxes in terms of Biot and Fourier numbers were highly similar and a dimensionless correlation was developed to quantify this heat transfer behavior, which showed that the ratio of the thermal resistance of the high temperature wall to the thermal resistance of convection heat transfer on the wall surface changed almost linearly during the process of spray-wall impingement.

## Introduction

In modern diesel homogeneous charge compression ignition (HCCI) engines, the phenomenon of spray-wall impingement is inevitable^1^. This causes the formation of fuel film over the piston surface, which affects the combustion, thermal efficiency, and emissions, as well as heat loss^2–4^. Transient heat transfer plays an important role in this process. The heat transfer in the spray-wall impingement influences the formation of wall film and the evaporation of fuel. In each cycle of the diesel engine, the heat transfer process of spray-wall impingement has different regimes including film evaporation, nucleate boiling, transition boiling and even film boiling^5^. The walls of the cylinder and piston are subjected to a wide range of surface heat flux ranging from zero to several MW/m^2^ ^6,7^. Therefore, measurement analysis of transient heat transfer of internal combustion engine can understand internal heat transfer processes to find ways to improve engine thermal efficiency and reduce emissions and heat loss^8^.

In recent years, researchers have conducted extensive studies on the heat transfer of spray-wall impingement for gasoline direct injection (GDI) engines^9–15^. Köpple et al.^9^ reported that a higher injection pressure resulted in a large surface temperature reduction but with low emissions due to less liquid film. Schulz et al.^10^ reported that the temperature at the impingement area highly decreased due to the transient heat transfer and was sensitive to spray angle, wall temperature and injection pressure. Serras-Pereira et al.^14,15^ reported that the heat flux during the spray-impingement was greatly related to the fuel temperature and properties. Relatively, only a few studies on the heat transfer of diesel engines have been published. Montanaro et al.^16^ found that an increase in wall temperature would reduce the spread of the liquid fuel. But when the wall temperature was higher than the Leidenfrost point, the liquid fuel started to spread again. Liu et al.^17^ found that cold wall temperature could inhibit the evaporation of the spray and the spread of the vapor phase along the surface. But as the wall temperature increased, an obvious improvement of the total vapor concentration could be observed. Li et al.^18^ reported that with the increase of the wall temperature, the volume of high-density region decreased until it disappeared, the dense droplets transferred to the periphery and the density distribution inclined to be uniform. Chen et al.^19^ reported that with the increase of the wall temperature, the vapor-rich field extended to the region near the wall and the area of vapor phase increased, but it had a little influence on the liquid phase of the spray. Du et al.^20^ reported that for a constant injection mass, higher injection pressure caused higher liquid-phase and vapor-phase spray diffusion rates. Park et al.^21^ reported that the increase in the injection pressure improved the fuel evaporation due to the superior atomization performance. Mahmud et al.^22^ reported that impingement was realized at faster rates with higher injection pressures due to the turbulent effect, which was reason for the high heat flux values. Li et al.^23^ studied the effect of ambient temperature on spray characteristics for large size marine engines. They reported that the ambient temperature had a slight influence on the vapor phase. However, it promotes the evaporation of the spray. Yu et al.^24^ reported that higher ambient temperatures accelerated the fuel droplet evaporation rate and so decreased the mass of impinged spray mass, resulting in decreases in impinged spray radius and height. In addition to the above factors mentioned, there are some other factors influencing the heat transfer of spray-wall impingement. For example, the nozzle hole diameter also has a strong influence on the heat transfer, as it affected the velocity of flame^22^. It could be seen that the above research is mainly based on the qualitative analysis of the changes in the spray structure caused by heat transfer, while the quantitative analysis by measuring the heat flux is relatively few.

The heat transfer of spray-wall impingement is a complicated and crucial process. At present, advanced heat insulation methods and high-pressure common-rail injection technology are employed to improve the thermal efficiency of the diesel engine, it leads to a further increase in injection pressure and piston surface temperature^25,26^. The increase in injection pressure can increase the momentum and the impact strength which enhances the heat transfer. However, the spray impinging on the superheated wall will reduce the heat transfer due to the film boiling effect^27–29^. In this study, the heat transfer characteristics of the spray-wall impingement were explored. A transient thermocouple was used to record the change in the wall surface temperature. The surface heat flux and heat transfer coefficient were calculated by one-dimensional calculation model and Newton's law of cooling, respectively. The heat transfer process was quantified by changing the experimental conditions such as injection pressure (40–160 MPa), initial wall temperature (200–600 °C) and ambient temperature (80–200 °C). The influence of different experimental conditions on heat transfer was explored by analysing the variations of heat transfer characteristics. The variation of dimensionless surface heat flux was discussed with respect to the dimensionless time by using Biot and Fourier numbers for different experimental conditions.

## Experimental setup and method

### Experimental setup

**Figure 1:**
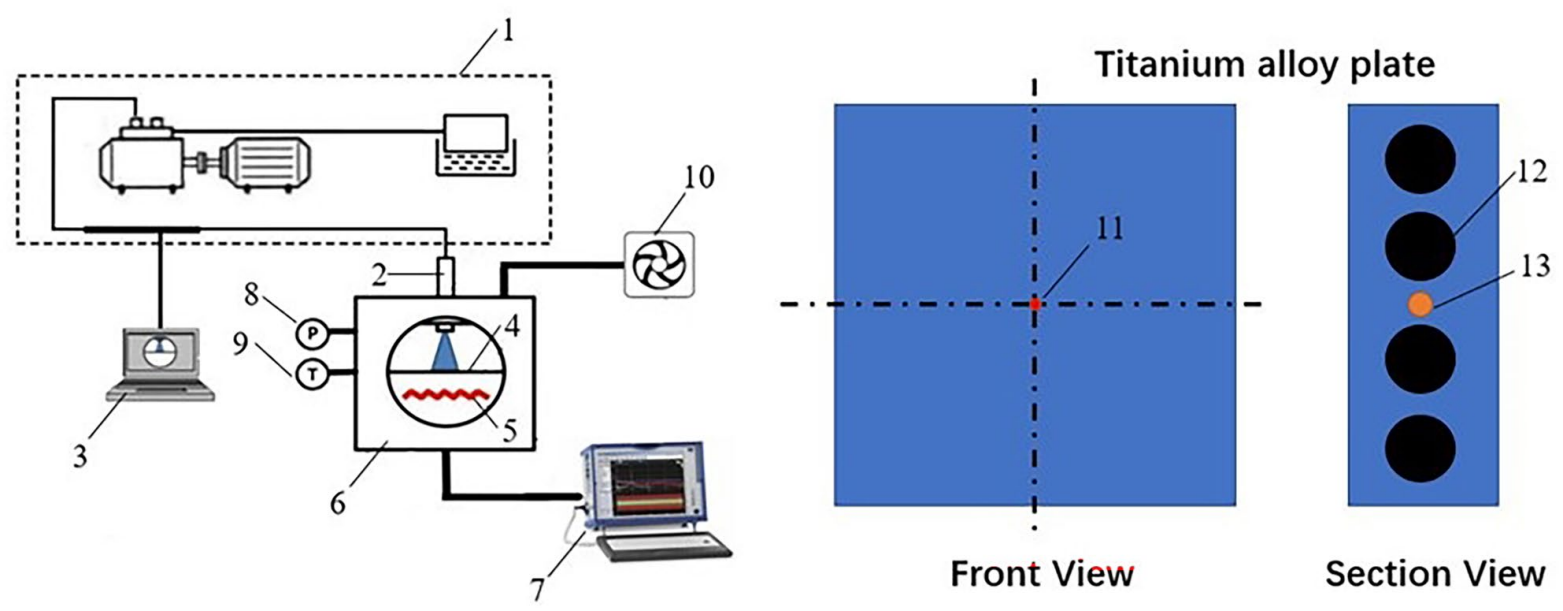
Schematic diagram of the spray-wall impingement test setup. (1-high-pressure common rail diesel injection system, 2-nozzle, 3-injector control system, 4-titanium alloy plate, 5-environmental heating system, 6-constant volume combustion chamber, 7-data acquisition system, 8-pressure sensor, 9- K-type thermocouple, 10-exhaust system, 11-transient thermocouple, 12-heating rod, 13-K-type thermocouple).

The schematic arrangement of the test setup is shown in Fig. 1. The tests were performed in a constant volume combustion chamber (CVCC). A single-hole injector of 0.26 mm diameter was located on the top of the chamber. The fuel was supplied through a high-pressure common rail diesel injection system. The desired injection pressure was created by a high-pressure oil pump driven by a variable frequency motor. The control of the fuel injector was realized by using self-made software on the computer. The chamber was open to the atmosphere and an exhaust system was connected with the constant volume combustion chamber to exhaust gas after each test.

Titanium alloy was selected as the material for high temperature wall because of its low thermal conductivity. A titanium alloy plate of size 100 mm × 100 mm × 20 mm was positioned downstream the injector nozzle and placed orthogonally to the injector axis. The thermal properties of the titanium alloy at different temperatures are shown in Table 1. The wall temperature could be changed from room temperature to 650 °C by four heating rods and monitored by a K-type thermocouple located in the center of the side of the plate. The charged gas in the CVCC could be heated to 220 °C by the ambient heating system and monitored by the same K-type thermocouple inserted into the inner wall of the chamber. Zhou et al.^30^ reported that only the temperature measured by the thermocouple located in the central of the spray-wall impingement changed significantly. In this study, a transient thermocouple (NANMAC-E6) with a response time of 20 microseconds was flush mounted at the center of the flat wall and parallel to the impingement surface to monitor the transient temperature of the wall surface during the process of spray impingement. A data acquisition system (DEWE-3020) was connected to the transient thermocouple to record the variation of wall surface temperature and the sampling frequency of the data acquisition system was set to 10,000 Hz. Therefore, the response time of the experimental system is 0.1 ms.

**Table 1:** Thermal properties of the titanium alloy at different temperatures.

Parameter	Value
**Thermal conductivity (W/(m °C))**	
200 °C	8.79
300 °C	10.47
400 °C	12.56
500 °C	14.24
600 °C	15.49
**Specific heat capacity (J/(kg °C))**	
200 °C	691
300 °C	703
400 °C	741
500 °C	754
600 °C	879
**Thermal diffusivity (m**^**2**^**/s)**	
200 °C	2.86 × 10^–6^
300 °C	3.35 × 10^–6^
400 °C	3.81 × 10^–6^
500 °C	4.24 × 10^–6^
600 °C	3.96 × 10^–6^
**Density (kg/m**^**3**^**)**	445

### Experimental conditions

In this experiment, 0^#^ diesel was used and the basic properties of the 0^#^ diesel and the experimental conditions are shown in Table 2. The ambient pressure and the impingement distance were 0.1 MPa and 50 mm. The fuel injection duration was kept at 3 ms. The experimental error was minimized by finding the average value of the measured parameter during three tests.

**Table 2:** Basic properties of 0^#^ diesel and experimental conditions.

Parameter	Value
Cetane number	45
Distillation range (°C)	180–370
Density (kg/m^3^, 20 °C)	848
50% distilled temperature (°C)	300
95% distilled temperature (°C)	365
Kinematic viscosity (mm^2^/s, 20 °C)	3.8
Ambient temperature (°C)	80/140/200
Injection pressure (MPa)	40/70/100/130/160
Initial surface temperature (°C)	200/300/400/450/500/550/600

### Surface heat flux calculation

Surface heat flux and heat transfer coefficient are two important parameters for characterizing heat transfer. In this study, the surface heat flux is calculated by using a one-dimensional finite difference heat conduction model which was based on the research of Reichelt et al.^31^. The use of the one-dimensional model provided the results with reasonable accuracy as the heat flux in the direction parallel to the wall surface was less than 10% of the heat flux in the normal direction^32^. The one-dimensional finite-difference conduction model is

1 $$\frac{\partial \theta }{{\partial \tau }} = \frac{{\partial^{2} \theta }}{{\partial \xi^{2} }}$$

2 $$\theta \left( {\xi ,\tau } \right) = T_{{\text{s}}} - T_{{{\text{init}}}} \quad \tau = \frac{\alpha t}{{L^{2} }}\quad \xi = \frac{z}{L}$$

where *T*_s_ is the surface temperature, *T*_init_ is the initial surface temperature, *t* is the time, $\tau$ is the dimensionless time, $\xi$ is the dimensionless coordinate, *z* is the normal coordinate, *L* is the thickness of the titanium alloy plate, and $\alpha$ is the thermal diffusivity.

It is assumed that the temperature distribution of the plate is uniform and the temperature of the bottom surface of the plate is constant due to its thickness and the duration of each experiment in the order of milliseconds. The boundary conditions and initial value conditions of Eq. (1) are shown in Eq. (3):

3 $$\left\{ \begin{gathered} \theta \left( {0,t} \right) = T_{{\text{s}}} (t) - T_{{{\text{init}}}} = \theta_{{\text{s}}} \left( t \right) \hfill \\ \theta \left( {1,t} \right) = 0 \hfill \\ \theta \left( {\xi ,0} \right) = 0 \hfill \\ \end{gathered} \right.$$

where $\lambda$ is the thermal conductivity, the transient surface heat flux of the impingement spray can be calculated by Eq. (4):

4 $$q_{{\text{s}}} = - \lambda \frac{\partial T}{{\partial z}}(0,t) = - \frac{\lambda }{L}\frac{\partial \theta }{{\partial \xi }}\left( {0,\tau } \right)$$

The above equation is transformed by using the Laplace transform and convolution theorem. The transient surface heat flux is computed at discrete time intervals $\tau_{i} = i \cdot \Delta \tau$ ($\tau < < 1$) by using Eq. (5):

5 $$q_{{\text{s}}} \left( {\tau_{i} } \right) = \frac{\lambda }{\sqrt \pi \cdot L}\sum\limits_{j = 0}^{i - 1} {\int_{{\tau_{j} }}^{{\tau_{j + 1} }} {\frac{{dT_{{\text{s}}} }}{{d\tau^{*} }}\left( {\tau^{*} } \right)} } \cdot \frac{1}{{\sqrt {\tau - \tau^{*} } }} \cdot d\tau^{*}$$

In order to obtain a good approximation of the interval [$\tau_{j} ,\tau_{j + 1}$], the Taylor series expansion is used:

6 $$\begin{aligned} & \frac{{dT_{{\text{s}}} }}{{d\tau^{*} }}(\tau^{*} ) = \frac{{dT_{{\text{s}}} }}{{d\tau^{*} }}(\tau_{{\text{M}}} ) + (\tau^{*} - \tau_{{\text{M}}} )\frac{{d^{2} T_{{\text{s}}} }}{{d\tau^{{*^{2} }} }}(\tau_{{\text{M}}} ) \\ & \tau_{{\text{M}}} = \frac{{\tau_{j} + \tau_{j + 1} }}{2} = \Delta \tau \frac{2j + 1}{2} \\ \end{aligned}$$

Using

7 $$\begin{aligned} & T_{{{\text{s}},j}}^{^{\prime}} = \frac{{dT_{{\text{s}}} }}{{d\tau^{*} }}(\tau_{{\text{M}}} ) = \frac{{T_{{{\text{s}},j + 1}} - T_{{{\text{s}},j}} }}{\Delta \tau } \\ & T_{{{\text{s}},j}}^{\prime \prime } = \frac{{d^{2} T_{{\text{s}}} }}{{d\tau^{{*^{2} }} }}(\tau_{{\text{M}}} ) = \frac{{\left( {T_{{{\text{s}},j + 2}} - T_{{{\text{s}},j + 1}} } \right) - \left( {T_{{{\text{s}},j}} - T_{{{\text{s}},j - 1}} } \right)}}{{2(\Delta \tau )^{2} }} \\ \end{aligned}$$

Equation (5) can be written as:

8 $$q_{{\text{s}}} \left( {\tau_{j} } \right) = \frac{\lambda }{\sqrt \pi \cdot L}\sum\limits_{j = 0}^{i - 1} {\int_{{\tau_{j} }}^{{\tau_{j + 1} }} {\left[ {(T_{{{\text{s}},j}}^{^{\prime}} + (\tau^{*} - \tau_{{\text{M}}} )T_{{{\text{s}},j}}^{^{\prime\prime}} )} \right]} } \cdot \frac{1}{{\sqrt {\tau_{i} - \tau^{*} } }} \cdot d\tau^{*}$$

Integrating Eq. (8) yields Eq. (9) to calculate the surface heat flux from the measured data:

9 $$\begin{aligned} & q_{{\text{s}}} \left( {\tau_{i} } \right) = 2\frac{\lambda }{L}\sqrt {\frac{\Delta \tau }{\pi }} \sum\limits_{j = 0}^{i - 1} {\left[ {\left( {T_{{{\text{s}},j}}^{^{\prime}} + T_{{{\text{s}},j}}^{\prime \prime } \Delta \tau \left( {i - \frac{2j + 1}{2}} \right)} \right) \cdot R_{i,j} - T_{{{\text{s}},j}}^{\prime \prime } \frac{\Delta \tau }{3}S_{i,j} } \right]} \\ & R_{i,j} = \left( {i - j} \right)^{1/2} - \left( {i - j - 1} \right)^{1/2} \\ & S_{i,j} = \left( {i - j} \right)^{3/2} - \left( {i - j - 1} \right)^{3/2} \\ \end{aligned}$$

**Figure 2:**
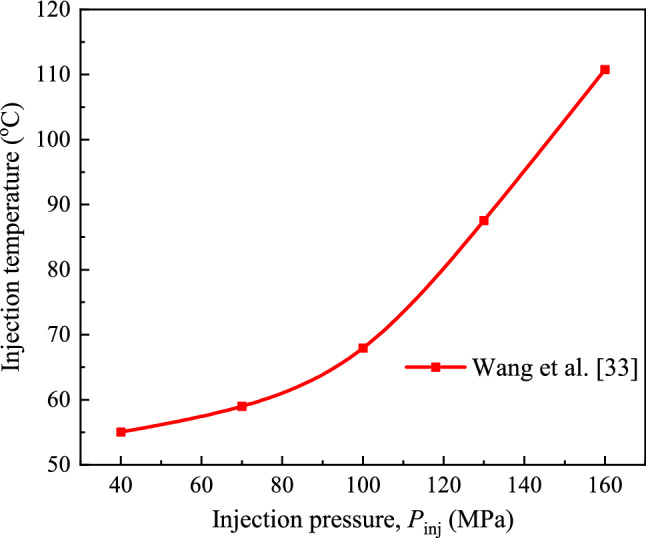
Injection temperature at the injector nozzle under different injection pressures.

The heat transfer coefficient is calculated by using Newton's law of cooling. Wang et al.^33^ reported that the friction between the fuel and the high-pressure fuel pipe and the throttling effect at the injector nozzle could increase the temperature of the fuel, and the increase was related to the injection pressure, as shown in Fig. 2.

Thus, the heat transfer coefficient can be calculated by Eq. (10):

10 $$h = \frac{{q_{{\text{s}}} }}{{\left( {T_{{\text{s}}} - T_{{{\text{inj}}}} } \right)}}$$

where *h* is the heat transfer coefficient, *T*_inj_ is the fuel temperature at the injector outlet.

It should be noted that in the process of moving from the injector outlet to the high temperature wall, the temperature of the fuel droplet will change due to the influence of the ambient temperature, but this temperature value is difficult to measure. In this experiment, the fuel temperature at the injector outlet (*T*_inj_) is used to calculate the equivalent heat transfer coefficient (*h*) for the heat transfer process of spray-wall impingement.

## Experimental uncertainty analysis

**Figure 3:**
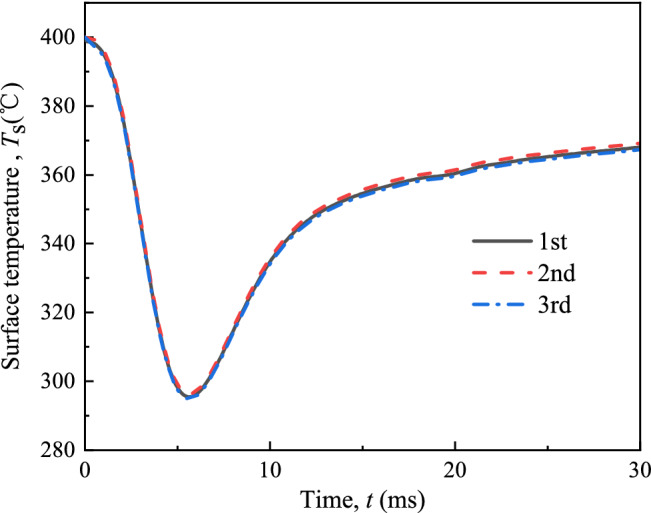
Three measurement results of the surface temperature (*T*_s_) under experimental conditions of *T*_init_ = 400 °C, *P*_inj_ = 160 MPa and *T*_amb_ = 80 °C.

As for the experimental error, the accuracy of the injection system and the two K-type thermocouples are ± 0.5 MPa and ± 1 °C respectively. For the transient thermocouple has been carefully calibration to obtain the correlations between the temperature and voltage. The accuracy of transient thermocouple is ± 0.1 °C. During three measurements of surface temperature at the same experimental conditions, the temperature curves are very close to each other, and the standard deviation of most measurement points are less than 2 °C. For example, under the experimental conditions that the injection pressure (*P*_inj_) was 160 MPa, the initial surface temperature (*T*_init_) was 400 °C, and the ambient temperature (*T*_amb_) was 80 °C, the three measurement results of the surface temperature (*T*_s_) are shown in Fig. 3. As a result, the surface heat flux (*q*_s_) and the heat transfer coefficient (*h*) based on surface temperature data are also highly repeatable. The standard deviation of *q*_s_ and *h* are less than 0.4 MW/m^2^ and 2 kW/(m^2^ °C) respectively. In the following results, error bars are shown in the curves of maximum heat flux (*q*_s,max_) and maximum heat transfer coefficient (*h*_max_), but not shown in the curves of *T*_s_, *q*_s_ and *h* because of a lot data points and small uncertainty.

## Results and discussions

### Effect of initial surface temperature

**Figure 4:**
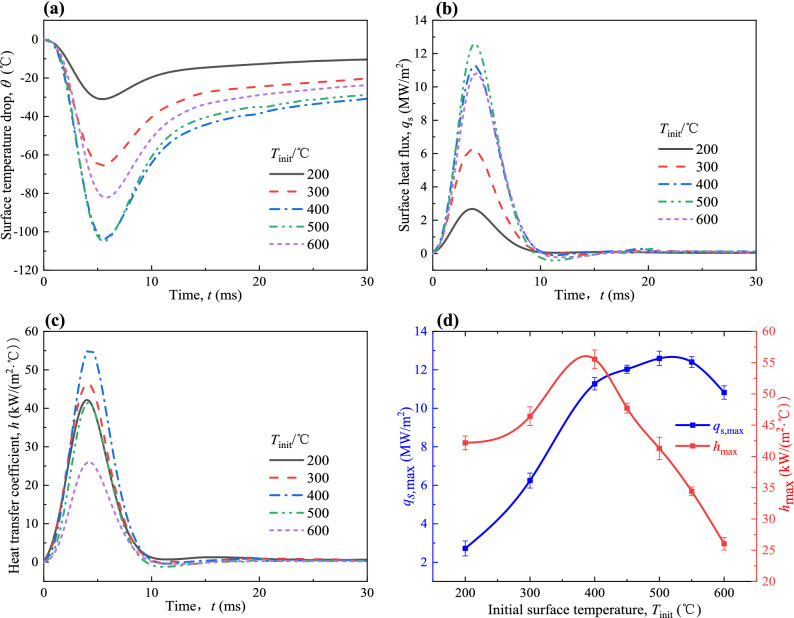
Variations of (**a**) *θ*, (**b**) *q*_s_, (**c**) *h*, and (**d**) *q*_s,max_ and *h*_max_ at different *T*_init_ with spray-wall impingement for *P*_inj_ = 160 MPa and *T*_amb_ = 80 °C.

The effect of initial surface temperature (*T*_init_) on the variations of surface temperature drop (*θ*), surface heat flux (*q*_s_) and heat transfer coefficient (*h*) for *P*_inj_ = 160 MPa and *T*_amb_ = 80 °C are shown in Fig. 4a–c. As shown in Fig. 4a, when the phenomenon of spray-wall impingement occurs, the surface temperature drops rapidly and reaches a minimum. After that, the surface temperature recovers at a slower rate. It can be seen from Fig. 4b,c that *q*_s_ and *h* both reach the peak rapidly and then decrease rapidly and finally stabilize. During the process of spray-wall impingement, the quick increase of *q*_s_ and *h* can be explained by the phenomenon of spray-wall impingement leading to a strong convection on the high temperature surface with a high local heat transfer coefficient^34^. However, the fuel will adhere to the wall and form a liquid film after the fuel injection is finished. At this time, evaporation from the liquid film surface or within the liquid film dominates the heat transfer on the wall surface, which is lower than the strong convection caused by spray-wall impingement. Under the combined effect of liquid film evaporation and wall heating, the surface temperature (*T*_s_) recovers slowly, *q*_s_ and *h* gradually decrease.

It can be seen from Fig. 4b,c that as *T*_init_ increases from 200 to 600 °C, the maximum surface heat flux (*q*_s,max_) and the maximum heat transfer coefficient (*h*_max_) first increase and then decrease. It is evident from the discussion that *T*_init_ has a considerable effect on the heat transfer. It can be found that *q*_s,max_ and *h*_max_ both have a turning point in the initial surface temperature range of 400–600 °C. In order to explore the variation of heat transfer in this temperature range, additional tests were performed by varying initial surface temperature at 450 °C and 550 °C. The resultant variations of *q*_s,max_ and *h*_max_ are shown in Fig. 4d.

It can be seen from Fig. 4d that the experimental error will not affect the variations of *q*_s,max_ and *h*_max_ with *T*_init_. When the *T*_init_ is less than 250 °C, the heat transfer on the wall is in a film evaporation state. As *T*_init_ increases from 250 to 390 °C, the temperature difference between the fuel droplets and high temperature surface gradually increases, more components in fuel droplets reach boiling point. It causes the gradual formation of vapour bubbles on the wall surface. The *q*_s,max_ and *h*_max_ increase rapidly, which can probably be attributed to the appearance of nucleate boiling. The *h*_max_ reaches the maximum value at about *T*_init_ of 390 °C. As *T*_init_ continues to increase, the evaporation becomes more intense and more bubbles are formed, which reduces the direct liquid contact with the wall and increases the thermal resistance between the fuel droplets and high temperature surface, the *h*_max_ tends to decrease gradually. However, *q*_s,max_ continues to increase slowly due to the bubbles taking away a large amount of latent heat of vaporization, and reaches the maximum value at *T*_init_ of 520 °C. With the further increase of *T*_init_, the heat transfer on the high temperature surface gradually enters the transition film boiling stage, which is accompanied by the formation of more bubbles^35,36^. It greatly increases the thermal resistance which leads to a decrease in the values of *q*_s,max_ and *h*_max_. From the above discussion, it can be concluded that *T*_init_ greatly affects the heat transfer during the spray-wall impingement and the variations of *q*_s,max_ and *h*_max_ are similar to that of pool boiling.

### Effect of injection pressure

**Figure 5:**
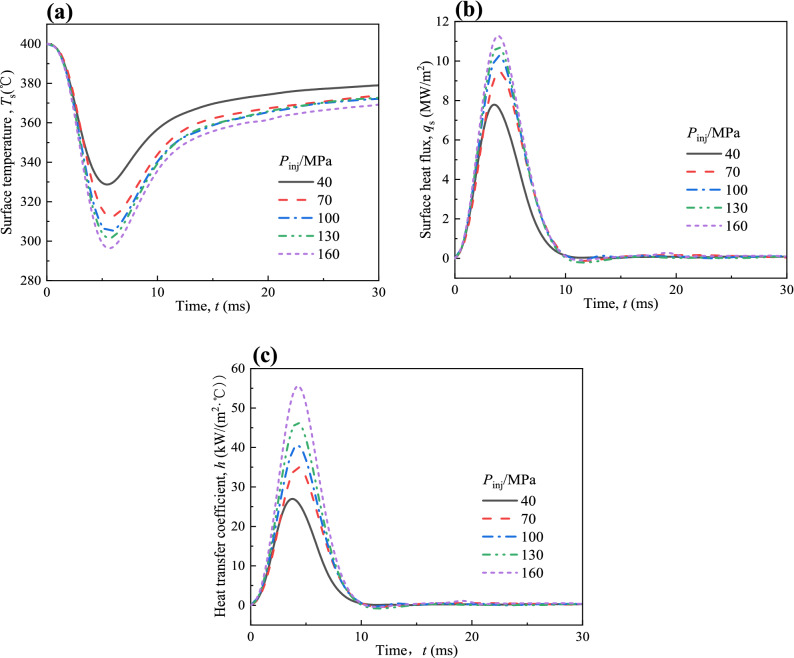
Variations of (**a**) *T*_s_, (**b**) *q*_s_ and (**c**) *h* at different *P*_inj_ with spray-wall impingement for *T*_init_ = 400 °C and *T*_amb_ = 80 °C.

The effect of injection pressure (*P*_inj_) on the variations of surface temperature (*T*_s_), surface heat flux (*q*_s_) and heat transfer coefficient (*h*) for *T*_init_ = 400 °C and *T*_amb_ = 80 °C are shown in Fig. 5a–c. As shown in Fig. 5a, *T*_s_ drops with time and this behavior becomes more obvious as *P*_inj_ increases, which leads to an increase in *q*_*s*_ and *h* as shown in Fig. 5b,c. A larger *P*_inj_ can increase the momentum of fuel droplets which helps to produce a stronger impingement that breaks the fuel film and enhances the heat transfer. As *P*_inj_ increases from 40 to 100 MPa and from 100 to 160 MPa, *q*_s,max_ increases 2.48 MW/m^2^ and 1.01 MW/m^2^, *h*_max_ increases 13.49 kW/(m^2^ °C) and 15.09 kW/(m^2^ °C), respectively. The increase in *q*_s,max_ becomes smaller, but the increase in *h*_max_ is basically same. This is because the increase of injection pressure can increase *T*_inj_, making fuel droplets easier to evaporate during the spraying process, thereby reducing the strength of the impingement. However, increasing *P*_inj_ can increase the spray velocity and increase the intensity of turbulence near the wall, thereby increasing the heat transfer effect on the high temperature surface^37^. From the above discussion, it can be concluded that the heat transfer of spray-wall impingement can be enhanced when fuel impinges with a large injection pressure on the wall.

### Effect of ambient temperature

**Figure 6:**
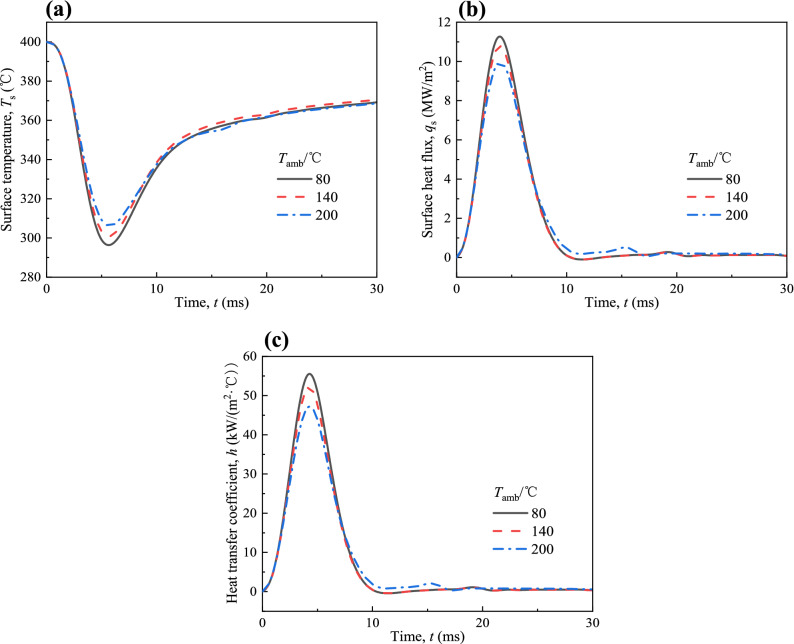
Variations of (**a**) *T*_s_, (**b**) *q*_s_ and (**c**) *h* at different *T*_amb_ with spray-wall impingement for *T*_init_ = 400 °C and *P*_inj_ = 160 MPa.

The effect of ambient temperature (*T*_amb_) on the variations of surface temperature (*T*_s_), surface heat flux (*q*_s_) and heat transfer coefficient (*h*) for *T*_init_ = 400 °C and *P*_inj_ = 160 MPa are shown in Fig. 6a–c. It is found that a larger *T*_amb_ causes a lower reduction of *T*_s_, a lower *q*_s_ and a lower *h*. As *T*_amb_ increases from 80 to 140 °C and from 140 to 200 °C, *q*_s,max_ decreases 0.49 MW/m^2^ and 0.81 MW/m^2^, *h*_max_ decreases by 3.41 kW/(m^2^ °C) and 4.91 kW/(m^2^ °C), respectively. The effect on the heat transfer of spray-wall impingement is smaller than *T*_init_ and *P*_inj_. Fuel droplets evaporate more easily at high ambient temperatures. The momentum of the fuel droplets decreases, which weakens the strength of the impingement, resulting in a decrease in the heat transfer of spray-wall impingement. In addition, the increase in *T*_amb_ also reduces the temperature difference between the fuel droplets and high temperature surface, allowing more fuel to evaporate after contacting the high temperature wall, thereby reducing the formation of the liquid film, which is beneficial to the performance of the engine. It can be concluded from above discussion that *T*_amb_ also has a measurable effect on the evaporation of fuel and the heat transfer of spray-wall impingement.

### Analysis of the effects of different experimental factors

Effect of the three experimental factors, namely the initial surface temperature (*T*_init_), injection pressure (*P*_inj_) and ambient temperature (*T*_amb_) on the heat transfer characteristics containing surface heat flux (*q*_s_) and heat transfer coefficient (*h*) are investigated separately. The effects of factors are summarized in Table 3. With the increase of *T*_init_, the heat transfer of spray-wall impingement first increases and then decreases. This variation can explain the phenomenon that the spread length of the fuel liquid phase decreased first and then increased with the increase of the wall temperature^16^. The increase in *P*_inj_ can improve the heat transfer of spray-wall impingement due to the increase in the strength of the impingement and the turbulence intensity on the wall, this variation confirms the phenomenon that the area of the fuel vapor phase increases with the increase of *P*_inj_^19^. The increase in *T*_amb_ accelerates the fuel droplet evaporation rate, decrease the mass of impinged spray and so weakens the strength of the impingement. It explains the phenomenon that as *T*_amb_ increases, *q*_s_ and the mass of wall liquid film decrease^38,39^.

**Table 3:** Effects of the different experimental factors on the heat transfer characteristics of spray-wall impingement.

	Surface heat flux	Heat transfer coefficient
Initial surface temperature *T*_init_ ↑	↑↓	↑↓
Injection pressure *P*_inj_ ↑	↑	↑
Ambient temperature *T*_amb_ ↑	↓	↓

↑: increase; ↓: decrease; ↑↓: first increase and then decrease.

The concept of a Contribution Index has been used to evaluate the contribution of the above three experimental factors on the heat transfer characteristics of spray-wall impingement^40^. The Contribution Index is calculated by Eq. (11):

11 $${\text{Contribution}}\;{\text{Index}} = \frac{{C_{1} }}{C} \times 100\%$$

*C* and *C*_1_ are the area between the *q*_s_ or *h* curves with time, which can be calculated by integrate the curves with time. *C* is obtained under the reference condition (Case 3, when *T*_init_ = 400 °C, *P*_inj_ = 160 MPa, *T*_amb_ = 80 °C), while *C*_1_ is obtained under other experimental condition. The black dotted line indicates the reference condition. If the value of the Contribution Index is greater than 100%, *q*_s_ and *h* have been increased, while the value of the Contribution Index is less than 100%, *q*_s_ and *h* have been decreased. The greater Contribution Index is, the greater the effect of the experimental factor on *q*_s_ and *h* has.

**Figure 7:**
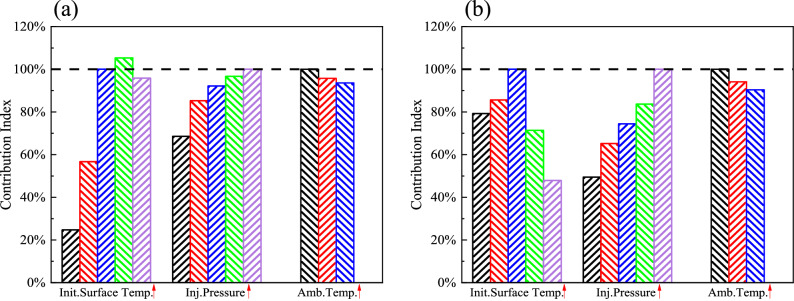
Contribution Indexes of different experimental factors (*T*_init_, *P*_inj_, *T*_amb_) on (**a**) *q*_s_ and (**b**) *h*.

The colors in the histogram in Fig. 7 correspond to the experimental conditions of the curve colors in Figs. 4, 5 and 6. The contribution indexes of the three experimental factors (*T*_init_, *P*_inj_, *T*_amb_) to the surface heat flux (*q*_s_) is shown in Fig. 7a. Under the conditions of this paper, it can be found the increase of *T*_init_ has a significant effect, the effect of increasing *P*_inj_ gradually decreases and the increase of *T*_amb_ has a minor effect. The contribution indexes of the three experimental factors (*T*_init_, *P*_inj_, *T*_amb_) to the heat transfer coefficient (*h*) is shown in Fig. 7b. The effect of increasing *T*_amb_ on *h* is similar to *q*_s_. Compared to the effect on *q*_s_, both *T*_init_ and *P*_inj_ have a significant effect on *h*. In summary, *T*_init_ has the most influential effect on the heat transfer of spray-wall impingement, followed by *P*_inj_ and *T*_amb_. It showed the conversion of heat transfer regime and the momentum of the spray are the main internal factors which determine the heat transfer characteristics of spray-wall impingement, which are mainly determined by *T*_init_ and *P*_inj_.

### Normalized surface heat fluxes at different experimental conditions

It can be concluded from the above results that the surface heat flux (*q*_s_) changes rapidly during the process of spray-wall impingement. Under all experimental conditions, *q*_s_ will increase rapidly and reach a peak after almost the same time interval. Subsequently, it decreases sharply until it reaches a steady state. Generally, the maximum surface heat flux (*q*_s,max_) and its corresponding time (*t*_max_) are considered to be important parameters for characterizing the transient heat transfer process^41–43^. Table 4 lists all the values of *q*_s,max_ and *t*_max_ at different experimental conditions. It can be found that *t*_max_ is approximately the same, which range from 3.6 to 4 ms, and it hardly depends on the experimental conditions. However, *q*_s,max_ is highly dependent on the experimental conditions.

**Table 4:** Values of the maximum surface heat flux (*q*_s,max_) and the time (*t*_max_) of its occurrence for various experimental conditions.

Exp. cases	*P*_inj_/(MPa)	*T*_init_/(°C)	*T*_amb_/(°C)	*q*_s,max_/(MW/m^2^)	*t*_max_/(ms)
1	160	**200**	80	2.68	3.6
2	160	**300**	80	6.24	3.7
3	160	**400**	80	11.27	3.9
4	160	**450**	80	12.03	3.8
5	160	**500**	80	12.59	3.9
6	160	**550**	80	12.41	3.9
7	160	**600**	80	10.82	4
8	**40**	400	80	7.79	3.6
9	**70**	400	80	9.44	4
10	**100**	400	80	10.26	4
11	**130**	400	80	10.72	3.9
3	**160**	400	80	11.27	3.9
3	160	400	**80**	11.27	3.9
12	160	400	**140**	10.79	3.9
13	160	400	**200**	9.97	4

Significant values are in [bold].

The dimensionless parameters such as Biot number (*Bi*^∗^) and Fourier number (*Fo*_s_), are introduced to describe the transient heat transfer process of spray-wall impingement. As an important dimensionless parameter in the analysis of heat conduction, *Bi*^∗^ represents the relative value of thermal resistance of conduction that of the convection thermal resistance in a given heat conduction system. *Fo*_s_ characterizes the dimensionless time in the one-dimensional unsteady heat transfer process. The expressions of *Bi*^∗^ and *Fo*_s_ are shown in Eq. (12) and Eq. (13), respectively.

12 $$Bi^{*} = \frac{h\delta }{\lambda } = \frac{{q_{{\text{s}}} \delta }}{{\left( {T_{{\text{s}}} - T_{{{\text{inj}}}} } \right)\lambda }}$$

13 $$Fo_{{\text{s}}} = \frac{\alpha t}{{\delta^{2} }}$$

where $\delta$, $\lambda$, $\alpha$ are the characteristic length, thermal conductivity coefficient, and thermal diffusivity coefficient of the wall. For a flat wall, the characteristic length is half of its thickness.

**Figure 8:**
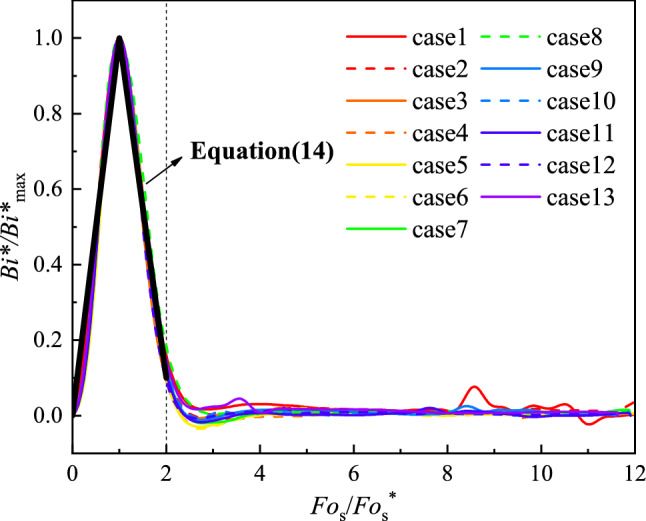
Non-dimensional surface heat flux versus dimensionless time.

We denote *Fo*_s_ corresponding to Bi^*^_max_ as *Fo*_s_^*^. The normalized relationship between the dimensionless surface heat flux (Bi^*^/Bi^*^_max_) and the dimensionless time (*Fo*_s_/*Fo*_s_^*^) for all experimental conditions is shown in Fig. 8. It showed that Bi^*^/Bi^*^_max_ significantly varies similarly for the *Fo*_s_/*Fo*_s_^*^ less than 2 under different conditions. Deviations in Bi^*^/Bi^*^_max_ curves can be seen for the *Fo*_s_/*Fo*_s_^*^ lying between 2 and 4. In the former, the generalized functional correlation of Bi^*^/Bi^*^_max_ and *Fo*_s_/*Fo*_s_^*^ can be expressed by two linear variations, as shown in Eq. (14).

14 $$\frac{{Bi^{*} }}{{Bi_{\max }^{*} }} = \left\{ \begin{gathered} \, k_{1} \frac{{Fo_{{\text{s}}} }}{{Fo_{{\text{s}}}^{*} }} + b_{1} , \, 0 < \frac{{Fo_{{\text{s}}} }}{{Fo_{{\text{s}}}^{*} }} \le 1 \hfill \\ \, k_{2} \frac{{Fo_{{\text{s}}} }}{{Fo_{{\text{s}}}^{*} }} + b_{2} , \, 1 < \frac{{Fo_{{\text{s}}} }}{{Fo_{{\text{s}}}^{*} }} \le 2 \hfill \\ \end{gathered} \right.$$

Through linear regression analysis of dimensionless curves under all experimental conditions, the values of *k*_1_, *b*_1_, *k*_2_, and *b*_2_ of each curve are obtained. The coefficient of determination (*R*^2^) and the values of *F*-test are used to measure the regression quality. In each regression line, the value of *R*^2^ is greater than 0.96 and the value of *F*-test is much greater than the corresponding critical value of *F*-test when the significance level is 0.05, which proves the high quality of linear regression. The relevant data of linear regression analysis is shown in the “Supplementary material”. Therefore, the average value and standard deviation of the regression line coefficient can be obtained, as shown in Table 5. This dimensionless relationship can be used to quantify the transient heat transfer behaviour in the process of spray-wall impingement. It also shows that the ratio of the thermal resistance of heat conduction in the high temperature wall to the thermal resistance of convection through the fluid boundary layer changes almost linearly with time in this process.

**Table 5:** Average values and uncertainty of the coefficients.

Coefficient	Average value	Standard deviation
*k*_1_	1.199	0.012
*b*_1_	− 0.127	0.021
*k*_2_	− 0.995	0.032
*b*_2_	2.057	0.028

## Conclusions

This paper seeks the influence of initial surface temperature, injection pressure, and ambient temperature on the heat transfer of diesel spray impingement on a high temperature wall. The conclusions can be summarized as follows.

1. With the increase of the initial surface temperature from 200 to 600 °C, the maximum surface heat flux and the maximum heat transfer coefficient first increase and then decrease, and reach the maximum at the initial surface temperature of about 520 °C and 390 °C, respectively. As the initial surface temperature increases, due to the continuous formation of bubbles on the wall, the heat transfer regime on the high temperature wall changes from film evaporation to nucleate boiling and then to transition boiling. This means that there is an optimal value for the initial surface temperature to maximize the heat transfer characteristics during the process of spray-wall impingement.
2. With the increase of the injection pressure from 40 to 160 MPa, the increase in momentum of the fuel droplets increases the surface heat flux, but its promoting effect is gradually weakened. Because increasing the injection pressure can increase the temperature of the fuel droplets, make the fuel droplets evaporate easier, and weaken the impact effect. In contrast, the heat transfer coefficient steadily increases due to the high injection pressure which enhances the turbulence intensity near the wall. With the increase of the ambient temperature from 80 to 200 °C, the decrease in surface heat flux and heat transfer coefficient is due to the evaporation of more droplets, which reduces the impact intensities of fuel droplets.
3. As for the heat transfer of spray-wall impingement, the initial surface temperature is the most influential factor, followed by the injection pressure and the ambient temperature, and the conversion of heat transfer regime and the momentum of the spray are the main internal influencing factors. The dimensionless curves of surface heat flux in terms of Biot (*Bi*^∗^) and Fourier (*Fo*_s_) numbers are highly similar. A dimensionless correlation is obtained to quantify the transient heat transfer behaviour, which shows that the ratio of the thermal resistance of heat conduction in the high temperature wall to the thermal resistance of convection heat transfer on the wall surface changes almost linearly during the process of spray-wall impingement in diesel engines.

In this study, a fundamental study of the heat transfer characteristics of the spray-wall impingement is carried out at an ambient pressure of 0.1 MPa. This study provides support for the analysis of the heat transfer mechanism and the optimization of the heat transfer model in the process of the spray-wall impingement in diesel engines. However, ambient pressure is also one of the important factors that will affect the atomization of fuel and the impact strength of fuel on the wall, we will conduct further research under the conditions closer to the real diesel engine.

## Supplementary Information

Supplementary Table S1.

## Data Availability

The datasets generated during and/or analyzed during the current study are available from the corresponding author on reasonable request.

## Abbreviations

*b*: Intercept
*Bi*^*^: Biot number
*Bi*^*^_max_: Maximum Biot number
*c*: Specific heat capacity [J/(kg °C)]
CVCC: Constant volume combustion chamber
*Fo*_s_: Fourier number
*Fo*_s_^*^: *Fo*_s_ corresponding to *Bi*^*^_max_
*h*: Heat transfer coefficient [kW/(m^2^ °C)]
*h*_max_: Maximum heat transfer coefficient [kW/(m^2^ °C)]
*k*: Slope
*L*: Thickness of the plate [m]
*P*_inj_: Injection pressure [MPa]
*q*_s_: Surface heat flux [MW/m^2^]
*q*_s,max_: Maximum surface heat flux [MW/m^2^]
*R*^2^: Coefficient of determination
*t*: Time [ms]
*t*_max_: Time corresponding to maximum surface heat flux [ms]
*T*_amb_: Ambient temperature [°C]
*T*_init_: Initial surface temperature [°C]
$T_{{{\text{inj}}}}$: Fuel temperature at the injector outlet [°C]
*T*_s_: Surface temperature [°C]
*z*: Normal coordinate
$$\alpha$$: Thermal diffusivity [m^2^/s]
$\Delta \tau$: Dimensionless time-step
$$\delta$$: Characteristic length [m]
$\theta$: Surface temperature drop [°C]
$$\lambda$$: Thermal conductivity [W/(m·°C)]
$$\xi$$: Dimensionless coordinate
$$\tau$$: Dimensionless time
