# Supplementary material for: Experimental investigation of heat transfer for diesel spray impingement on a high temperature wall
Source: Sci Rep. 2022 Apr 26;12:6771. doi: 10.1038/s41598-022-10959-6 (PMC9042839; doi:10.1038/s41598-022-10959-6)
Supplement: Supplementary file 1 — Supplementary Table S1. [file 41598_2022_10959_MOESM1_ESM.docx]

**Experimental investigation of heat transfer for diesel spray impingement on a high temperature wall**

**Zhenyao Guo, Weizheng Zhang*, Shuang Jin, Zhicheng Shi & Yanpeng Yuan**

**School of Mechanical Engineering, Beijing Institute of Technology, Beijing, 100081, China.**

***Corresponding author email: zhangwz@bit.edu.cn**

**Supplementary Table S1** The coefficient of regression line and the values of *R*^2^ and *F* under each experimental condition.

| Exp.cases | *k*_1_ | *b*_1_ | *R*_1_^2^ | *F*_1_ | *k*_2_ | *b*_2_ | *R*_2_^2^ | *F*_2_ |
| --- | --- | --- | --- | --- | --- | --- | --- | --- |
| 1 | 1.167 | -0.079 | 0.99 | 2943.99 | -0.954 | 2.021 | 0.99 | 4801.15 |
| 2 | 1.196 | -0.098 | 0.98 | 2224.54 | -0.992 | 2.051 | 0.99 | 4091.75 |
| 3 | 1.201 | -0.135 | 0.98 | 1864.81 | -1.031 | 2.080 | 0.99 | 3862.66 |
| 4 | 1.197 | -0.143 | 0.97 | 1547.04 | -1.025 | 2.091 | 0.99 | 4527.52 |
| 5 | 1.198 | -0.155 | 0.97 | 1291.03 | -1.021 | 2.093 | 0.99 | 4757.67 |
| 6 | 1.214 | -0.143 | 0.97 | 1496.69 | -0.998 | 2.059 | 0.99 | 5839.99 |
| 7 | 1.207 | -0.141 | 0.98 | 1608.83 | -0.982 | 2.048 | 0.99 | 6035.27 |
| 8 | 1.215 | -0.116 | 0.98 | 1661.77 | -0.922 | 2.006 | 0.99 | 3383.63 |
| 9 | 1.201 | -0.135 | 0.98 | 1813.19 | -0.995 | 2.054 | 0.99 | 5271.49 |
| 10 | 1.189 | -0.126 | 0.98 | 2095.81 | -0.999 | 2.067 | 0.99 | 4732.63 |
| 11 | 1.196 | -0.136 | 0.98 | 1790.31 | -1.012 | 2.079 | 0.99 | 4546.72 |
| 12 | 1.205 | -0.133 | 0.98 | 1884.01 | -1.027 | 2.073 | 0.99 | 3934.11 |
| 13 | 1.195 | -0.116 | 0.98 | 2387.94 | -0.972 | 2.021 | 0.99 | 3850.93 |

*F*_0.05_(1,11) =4.84
